# Heart failure evidence update 2026

**DOI:** 10.1007/s10741-026-10609-3

**Published:** 2026-03-11

**Authors:** Sotiria Liori, Chris J. Kapelios, Gianluigi Savarese, Gerasimos Filippatos

**Affiliations:** 1 Heart Failure Unit, Department of Cardiology, School of Medicine, Attikon University Hospital, National and Kapodistrian University of Athens, Rimini 1, Haidari, Athens, 12462 Greece; 2 Department of Clinical Science and Education, Karolinska Institutet, Södersjukhuset, Stockholm, Sweden

**Keywords:** Heart failure, Pharmacotherapy, Outcomes, Clinical trials, Medical devices

## Abstract

The interval since the 2021 European Society of Cardiology (ESC) Guidelines and 2022 American Heart Association/American College of Cardiology/Heart Failure Society of America (AHA/ACC/HFSA) Guidelines publications has witnessed an unprecedented volume of evidence that substantially expands the therapeutic landscape across the entire ejection fraction (EF) spectrum. Major developments include the emergence of non-steroidal mineralocorticoid receptor antagonists and incretin-based therapies for heart failure (HF) with mildly reduced and preserved EF, the validation of rapid guideline-directed medical therapy optimization strategies in acute HF, and new evidence supporting digitalis glycosides in HF patients with reduced EF. Device-based care has evolved with transcatheter edge-to-edge repair for valvular heart disease. These data are likely to reshape contemporary clinical practice.

## Introduction

**Fig. 1:**
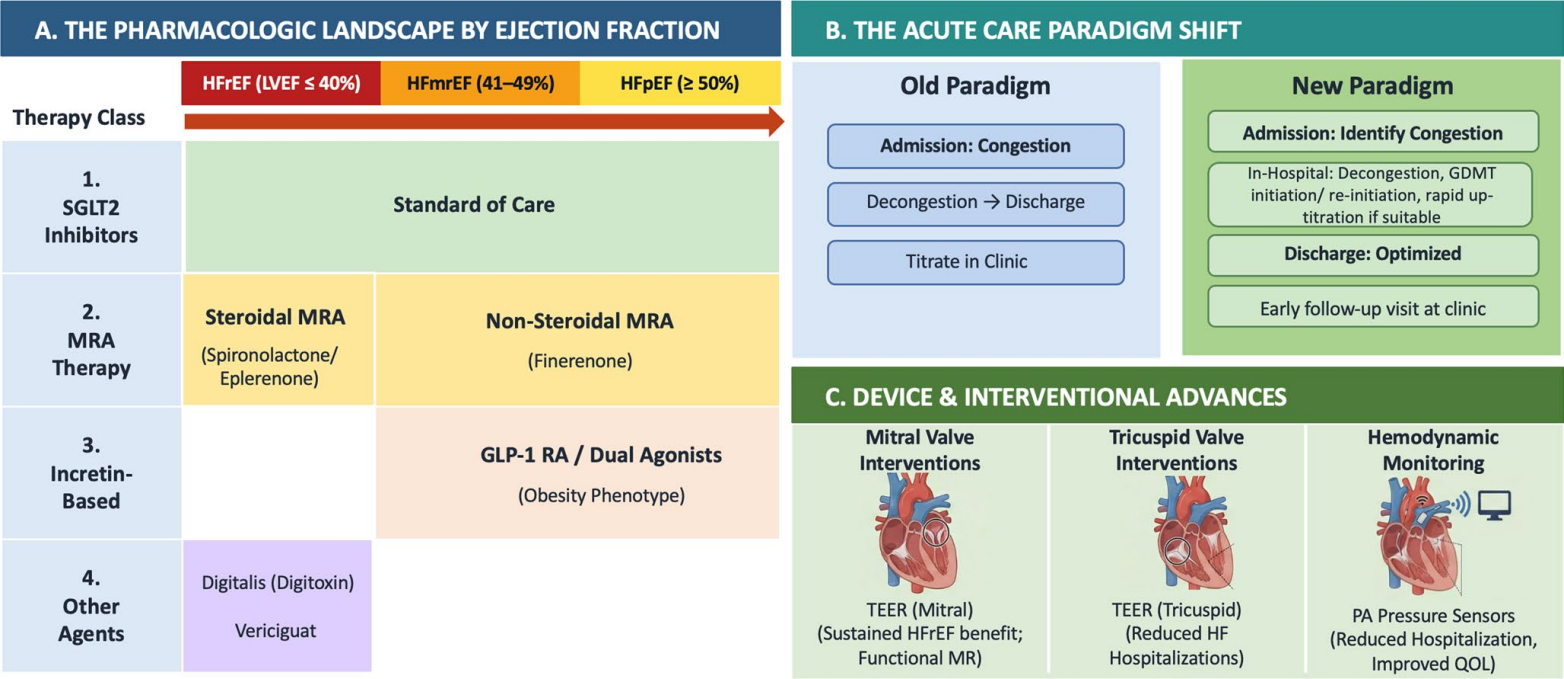
Central Illustration: The Evidence Surge in Heart Failure Management. ARNI = Angiotensin Receptor-Neprilysin Inhibitor; BB = Beta-Blocker; EF = Ejection Fraction; GDMT = Guideline-Directed Medical Therapy; HF = Heart Failure; MRA = Mineralocorticoid Receptor Antagonist; PA = Pulmonary Artery; QOL = Quality of Life; SGLT2i = Sodium-Glucose Cotransporter-2 Inhibitor; TEER = Transcatheter Edge-to-Edge Repair

In the years following the latest European and US practice guidelines on the management of heart failure (HF), we have witnessed a remarkable bloom in evidence generation, calling for an updated synthesis of the best available data to inform clinical practice. Multiple phase 3 randomized controlled trials (RCTs) have reported results establishing therapeutic efficacy across the HF spectrum. Importantly, landmark trials reported in 2024 and 2025 established efficacy of non-steroidal mineralocorticoid receptor antagonists (MRAs) [1], and incretin-based therapies in selected patients with mildly reduced (HFmrEF) and preserved ejection fractions (HFpEF) [2, 3], populations with limited guideline-directed medical options until recently. **(**Fig. 1**)**

Concurrently, the 2025 Canadian Cardiovascular Society/Canadian Heart Failure Society (CCS/CHFS) Guidelines and the 2025 Japanese Circulation Society/Japanese Heart Failure Society (JCS/JHFS) Guidelines have incorporated these advances, providing updated algorithms for HF with nonreduced ejection fraction (HfnrEF; defined as a composite of HFmrEF [LVEF 41–49%] and HFpEF [LVEF ≥ 50%]), while transcatheter edge-to-edge repair (TEER) has extended from mitral to tricuspid regurgitation (TR) with validated clinical benefit [4–7]. 

In this report we aim to identify how these data confirm, refine, or challenge existing guideline recommendations and their anticipated impact on clinical practice.

## Acute heart failure: rapid optimization and decongestion strategies

Several new studies have provided evidence on the ongoing quest of defining and achieving optimal decongestion in the scenario of acute heart failure (AHF) with volume overload. The PUSH-AHF trial evaluated a strategy of guiding decongestive therapy using spot urine sodium [8]. While the study supports the clinical value of natriuresis-guided management, it is also notable that patients in the intervention arm received substantially higher loop diuretic doses than those in the control arm, suggesting that timely and adequate escalation of diuretic therapy -when response is insufficient- is the key component of effective decongestion. Point-of-care (handheld) sodium testing may help facilitate rapid treatment adjustments in this setting, and when tailored with a diuretic titration protocol may improve decongestion, as demonstrated by the small, single-centre, randomized, open-label EASY-HF trial [9]. 

Real-world data show that even fundamental, evidence-based decongestion steps, such as adequate initial intravenous (IV) loop diuretic dosing are inconsistently implemented [10]. Despite relevant evidence, there remains a substantial gap between recommendations and routine clinical practice, pertinent to physician and system-related barriers [11]. Thus, although the science supports rapid optimization and precision decongestion, translating this into standard of care remains challenging.

The DICTATE-AHF trial (*N* = 240) investigated early initiation of dapagliflozin in patients hospitalized with AHF. Dapagliflozin significantly enhanced natriuresis (median 50 vs. 35 mmol/40 mg IV furosemide, *p* = 0.025) and diuresis (median 634 vs. 403 mL/40 mg IV furosemide, *p* = 0.005) compared with usual care [12]. The DAPA-RESIST trial (*N* = 61) compared dapagliflozin vs. metolazone in patients hospitalized for HF and diuretic resistance, demonstrating similar efficacy with a potentially more favorable renal safety profile for dapagliflozin [13]. The EMPULSE trial (*n* = 530), although not a decongestion study, demonstrated that empagliflozin initiated in-hospital for AHF led to greater weight loss and more favorable weight change per mean daily loop diuretic dose through 90 days [14]. Notably, EMPULSE also showed improved clinical benefit (win ratio–based hierarchical composite of all-cause death, HF events/time to first event, and ≥ 5-point KCCQ improvement at 90 days). These trials suggest that in-hospital initiation of Sodium-Glucose Cotransporter-2 (SGLT2) inhibitors is safe, does not increase the risk of hypotension or renal injury, and could form a standard component of the initial AHF management bundle.

The CLOROTIC trial randomized 230 patients with chronic HF on low-to-moderate dose oral furosemide (80–240 mg/day) admitted for acute decompensation to 5 days of hydrochlorothiazide or placebo [15]. Hydrochlorothiazide significantly improved weight loss and weight change per 40 mg furosemide equivalent. However, increases in serum creatinine were more common with hydrochlorothiazide. A recent small RCT [16], evaluated 6-hour natriuresis in patients hospitalized with HF and diuretic resistance (6-hour cumulative natriuresis < 100 mmol after IV loop diuretic), randomizing them to either IV loop diuretic dose intensification (2.5x) or addition of IV chlorothiazide on top of the same loop diuretic dose, and found that both strategies improved natriuresis, with a significantly greater increase with chlorothiazide (treatment effect + 58 mmol; *P* < 0.001). These results strengthen the argument for earlier sequential nephron blockade among AHF patients with poor initial response to IV loops. Nonetheless, a design including bid IV administration of loops and a 24-hour outcome would have been more appropriate to account for the shorter t_1/2_ of loops relative to thiazides.

The ADVOR trial was a randomized, double-blind study of 519 patients with AHF, which compared the addition of IV acetazolamide to a placebo [17]. Despite limitations in its design, such as the restriction on early loop diuretic up-titration and the exclusion of patients taking SGLT2 inhibitors [18], a secondary analysis showing superior decongestive effects of IV acetazolamide on top of low-to-moderate dose intravenous loop diuretics in AHF patients with serum HCO3 above 27 mmol/l implies the potential utility of this approach to manage patients with diuretic resistance associated with metabolic alkalosis and impaired serum chloride signaling [19]. 

Management of AHF has shifted over the last years from focusing solely on symptomatic decongestion to the rapid initiation of disease-modifying therapies during hospitalization. Recent position papers have proposed that the hospitalization period be utilized not just for decongestion, but for the rapid initiation of guideline-directed medical therapy (GDMT), and guidelines have adopted this approach [20, 21]. A secondary analysis of STRONG-HF showed that rapid GDMT initiation and uptitration had a significant decongestive and diuretic sparing effect, likely shifting the decongestion paradigm from diuretic- to GDMT-centered [22, 23]. 

### SGLT2i: therapy across the ejection fraction spectrum

The most transformative development since 2021 has been the definitive establishment of SGLT2 inhibitors as foundational therapy for HF regardless of EF. While DAPA-HF and EMPEROR-Reduced had established efficacy in HF with reduced EF (HFrEF) prior to 2021, the EMPEROR-Preserved and DELIVER trials provided the evidence base to update the treatment indication of these agents in non-reduced EFs. Importantly, a post-hoc analysis from DELIVER demonstrated that dapagliflozin reduced cardiovascular (CV) death in the HF with improved EF (HFimpEF) subgroup (HR 0.62, 95% CI 0.41–0.96), supporting the utilization of SGLT2i in this unique patient population [24]. Meta-analyses combining these trials with earlier HFrEF studies confirm consistent benefits across all HF phenotypes, likely involving mechanisms beyond glycemic control and diuresis, including favorable myocardial metabolic effects, reduction in ventricular stiffness, and anti-inflammatory actions [25, 26]. 

The evidence base for SGLT2 inhibitors has extended into the post-myocardial infarction (MI) setting. The EMPACT-MI trial evaluated empagliflozin in patients with acute MI who had either new left ventricular ejection fraction (LVEF) < 45% or signs of congestion [27]. While the primary composite endpoint of time to first HF hospitalization or all-cause mortality was not significantly reduced (HR 0.90, 95% CI 0.76–1.06), empagliflozin significantly reduced first HF hospitalizations by 23% (HR 0.77) and total HF hospitalizations by 33% (RR 0.67). The DAPA-MI trial, evaluating dapagliflozin in patients with acute MI without prior diabetes or HF, did not meet significance for CV death or HF hospitalization but demonstrated significant benefits in preventing new-onset type 2 diabetes mellitus (T2DM) and sustained weight loss [28]. However, in the context of a neutral primary endpoint, these secondary findings should be interpreted cautiously and viewed as supportive/hypothesis-generating.

## Non-steroidal mineralocorticoid receptor antagonists

The FINEARTS-HF trial represents a landmark achievement for HFmrEF and HFpEF [1]. This study randomized 6,001 patients with HF and LVEF ≥ 40% to finerenone, a non-steroidal selective MRA, or placebo. Finerenone significantly reduced the composite primary endpoint of CV death and total worsening HF events by 16% (rate ratio [RR] 0.84, 95% CI 0.74–0.95, *p* = 0.007), representing an absolute rate reduction of 2.8 events per 100 patient-years [1]. Total HF events were reduced by 18% (RR 0.82, 95% CI 0.70–0.94, *p* = 0.006). Critically, the benefit of finerenone was consistent across the entire EF range studied, including those with EF ≥ 60%, a subgroup where prior trials of steroidal MRAs had shown attenuated or neutral efficacy. Safety analyses from FINEARTS-HF addressed longstanding concerns regarding hyperkalemia with MRA therapy. While hyperkalemia events with serum potassium > 5.5 mmol/L were more frequent in the finerenone arm (14.3% vs. 6.9%), the rate of hyperkalemia-related hospitalization remained low at 0.5% and did not negate clinical benefit. This trial provides the first robust evidence that MRA blockade improves outcomes in HFpEF, likely establishing finerenone as a second pillar of therapy for this phenotype alongside SGLT2 inhibitors. These findings are further supported by a 2024 individual patient-level meta-analysis of over 13,000 patients, which demonstrated that MRAs reduce the risk of CV death or HF hospitalization across the entire spectrum of EF [29]. The 2025 CCS/CHFS and JCS/JHFS guidelines now include MRA recommendations for symptomatic HFpEF and HFmrEF patients [4, 5]. 

FINE-HEART [30] was a prespecified, participant-level pooled analysis aggregating data from three Phase III randomized trials: FINEARTS-HF [1], FIDELIO-DKD [31], and FIGARO-DKD [32]. This pooling resulted in a dataset of 18,991 patients. The primary endpoint was defined as time to CV death, analyzed using stratified Cox proportional hazards models. The divergence between the primary analysis (*p* = 0.076) and the sensitivity analysis (*p* = 0.025) suggests that the neutral primary result was likely an artifact of the adjudication definition-as the primary analysis definition excluded deaths of undetermined cause-rather than a lack of biological efficacy. In a population with cardiovascular-kidney-metabolic syndrome (HF, chronic kidney disease [CKD], diabetes), the probability that an “undetermined” death being cardiovascular is extremely high. By excluding these deaths, the primary analysis likely discarded valid signal events. When these deaths were included, the 12% relative risk reduction became statistically significant. This provides suggestive evidence that finerenone may reduce the risk of CV death. It will be interesting to see how regulatory authorities and practice guideline committees will evaluate and handle these results. Additional evidence regarding steroidal MRAs is anticipated from the SPIRRIT-HFpEF and SPIRIT-HF [33, 34]. 

## Incretin-based therapies: targeting the obesity-heart failure phenotype

Obesity represents a major driver of HFpEF, creating a distinct phenotype characterized by plasma volume expansion, systemic inflammation, epicardial adipose tissue accumulation, and mechanical restraint of ventricular filling [35]. The STEP-HFpEF trial evaluated semaglutide 2.4 mg, a glucagon-like peptide-1 receptor agonist (GLP-1 RA), in 529 patients with HFpEF, BMI ≥ 30 kg/m², and no diabetes [3]. At 52 weeks, semaglutide demonstrated improvements in Kansas City Cardiomyopathy Questionnaire (KCCQ) clinical summary score (estimated difference 7.8 points, 95% CI 4.8–10.9, *p* < 0.001), body weight (estimated difference 10.7% points, 95% CI -11.9 to -9.4, *p* < 0.001) and Six-minute walk distance (6MWD, estimated difference 20.3 m, 95% CI 8.6–32.1, *p* < 0.001). Additionally, semaglutide significantly reduced serum C-reactive protein (CRP) values and NT-proBNP levels [3]. As weight loss typically raises natriuretic peptide concentrations, this reduction implies a distinct hemodynamic benefit beyond the effects of weight loss alone.

The STEP-HFpEF DM trial, which enrolled 1,145 patients with HFpEF, obesity, and type 2 diabetes mellitus (T2DM), demonstrated similar benefits, with pooled analysis of both trials showing a between-group difference of 7.5 points (95% CI 5.3–9.8, *p* < 0.0001) in KCCQ-CSS and 8.4% greater weight reduction (95% CI -9.2 to -7.5, *p* < 0.0001) at 52 weeks [36]. 

In a pooled participant-level analysis of the SELECT, FLOW, STEP-HFpEF, and STEP-HFpEF DM trials (*n* = 3,743) [37] over a 52-week period, patients treated with semaglutide 2.4 mg experienced a significant reduction in the composite endpoint of cardiovascular death or worsening HF events (HR 0.69; 95% CI 0.53–0.89; *p* = 0.0045). This benefit was driven predominantly by a lower incidence of worsening HF events (HR 0.59; 95% CI 0.41–0.82; *p* = 0.0019), while the reduction in CV death did not reach statistical significance (HR 0.82; 95% CI 0.57–1.16).

The SUMMIT trial evaluated tirzepatide, a dual glucose-dependent insulinotropic polypeptide (GIP) and GLP-1 receptor agonist, in 731 patients with HFpEF and BMI ≥ 30 kg/m² [2]. Tirzepatide significantly reduced the composite primary endpoint of CV death or worsening HF events by 38% vs. placebo (HR 0.62, 95% CI 0.41–0.95, *p* = 0.026). The magnitude of benefit positions tirzepatide as a therapy for the obese HFpEF phenotype, moving beyond simple weight management and quality of life improvement to direct CV risk reduction. The 2025 CCS/CHFS guidelines now recommend evidence-based drugs with GLP-1 receptor agonist activity for symptomatic patients with LVEF ≥ 45% and BMI ≥ 30 kg/m² [4]. 

The primary distinction between GLP-1 receptor agonists and dual GLP-1/GIP receptor agonists lies in their target engagement. While GLP-1 mono-agonists like semaglutide stimulate a single incretin pathway to regulate satiety and insulin secretion [38], dual agonists like tirzepatide simultaneously activate both GLP-1 and GIP receptors [39, 40]. This synergistic effect results in superior efficacy, with head-to-head trials demonstrating that dual agonists provide significantly greater reductions in both hemoglobin A1C (HbA1c) and body weight compared to selective GLP-1 inhibition in patients with T2DM, while maintaining a similar safety profile consisting mainly of gastrointestinal adverse events [41]. Complementing these metabolic improvements, imaging substudies suggest favorable effects on cardiac remodeling, with semaglutide demonstrating attenuation of left atrial and right ventricular enlargement and tirzepatide significantly reducing left ventricular mass and paracardiac adipose tissue volumes [42, 43]. 

## Digitalis glycosides: new evidence in contemporary practice

Based on the DIG trial and subsequent analyses [44], digoxin had a neutral effect on all-cause mortality but significantly reduced hospitalizations for worsening HF. Notably, benefits were most pronounced in high-risk subgroups (such as those with New York Heart Association [NYHA] class III-IV symptoms or LVEF < 25%) [45], and serum digoxin concentrations 0.5–0.9 ng/ml were associated with reduced mortality and hospitalizations [46]. The latest trial, DIGIT-HF, focused on a sicker population with advanced HF who were already well-treated with GDMT.

The DIGIT-HF trial evaluated digitoxin in 1,212 patients with chronic HFrEF (LVEF ≤ 40% with NYHA II or LVEF ≤ 30% with NYHA III-IV) on evidence-based HF therapy for ≥ 6 months [47]. This investigator-initiated, multicenter, randomized, double-blind, placebo-controlled, event-driven phase IV trial randomized patients to digitoxin 0.07 mg daily (titrated to target serum concentration 8–18 ng/ml) or placebo. At median follow-up of 36 months, digitoxin significantly reduced the primary endpoint of death from any cause or first hospitalization for HF by 18% (HR 0.82, 95% CI 0.69–0.98, *p* = 0.03), with an absolute risk reduction of 4.6% and number-needed-to-treat of 22.

The study population was on well-implemented contemporary HF therapy: 95% on beta blockers (BBs), 93% on renin–angiotensin system inhibitor (RASi [40% angiotensin receptor/neprilysin inhibitor -ARNI]), 76% on MRAs, 19% on SGLT2 inhibitors, 64% with implantable cardioverter defibrillator (ICD), and 25% with cardiac resynchronization therapy (CRT). Although digitoxin met criteria for noninferiority for mortality (HR margin 1.303, *p* < 0.001 for noninferiority), the individual components showed consistent directional benefit: all-cause death (HR 0.86, 95% CI 0.69–1.07) and first HF hospitalization (HR 0.85, 95% CI 0.69–1.05). These results suggest that digitalis glycosides may provide benefit in contemporary HFrEF patients already receiving foundational therapy. Given the positive reduction in the composite of death or HF hospitalization, this therapy is expected to represent an additional option for patients with advanced heart failure.

## Additional pharmacologic agents in heart failure with reduced ejection fraction

The VICTORIA trial had established vericiguat, a soluble guanylate cyclase (sGC) stimulator, in patients with worsening HFrEF, demonstrating a 10% reduction in the composite of CV death or first HF hospitalization (HR 0.90, 95% CI 0.82–0.98, *p* = 0.02) [48]. The VICTOR trial enrolled 6,105 ambulatory patients with HFrEF (LVEF ≤ 40%), elevated NT-proBNP (up to 6,000 pg/mL), and no recent HF events [49]. At median follow-up of 18.5 months, the primary endpoint of CV death or first HF hospitalization was not significantly reduced (HR 0.93, 95% CI 0.83–1.04, *p* = 0.22). However, prespecified secondary analyses showed significant reductions in CV death (HR 0.83, 95% CI 0.71–0.97, *p* = 0.02) and all-cause death (HR 0.84, 95% CI 0.74–0.97, *p* = 0.02) [49]. This again raises the question of how we handle significant changes in mortality in the absence of a non-significant change in the primary composite endpoints. In exploratory analyses, overall worsening HF (incorporating outpatient events and HF hospitalizations) was reduced (HR 0.90, 95% CI 0.81-1.00, *p* = 0.047).

Pooled analysis of VICTORIA and VICTOR (*n* = 11,155) demonstrated significant reductions in the primary composite (HR 0.91, 95% CI 0.85–0.98, *p* = 0.0088 [ARR 1.99%, NNT = 51]), CV death (HR 0.89, 95% CI 0.80–0.98, *p* = 0.020), HF hospitalization (HR 0.92, 95% CI 0.84-1.00, *p* = 0.043), and all-cause death (HR 0.90, 95% CI 0.82–0.99, *p* = 0.025), without statistically significant heterogeneity across the two trials [50]. 

Omecamtiv mecarbil, a selective cardiac myosin activator, was evaluated in the GALACTIC-HF trial in 8,256 patients with HFrEF with LVEF ≤ 35% [51]. The primary outcome of CV death or first HF event was reduced by a statistically significant 8% (HR 0.92, 95% CI 0.86–0.99, *p* = 0.03) but the benefit was not regarded clinically adequate to lead to regulatory approval (NNT 48) .Benefit was concentrated in patients with EF ≤ 28%, those with lower systolic blood pressure (SBP) ≤ 100 mmHg, and more severe HF [52, 53]. The ongoing COMET-HF trial is testing omecamtiv mecarbil in a refined, higher-risk population with severe LV dysfunction (LVEF < 30% [< 25% with AF]) and recent HF hospitalization or HF event [54]. 

## Intravenous iron therapy: refining patient selection

The management of iron deficiency (ID) in HF has evolved toward more precise patient selection. The IRONMAN trial randomized 1,137 patients with HFrEF with LVEF ≤ 45% and ID (transferrin saturation [TSAT] < 20% or ferritin < 100 µg/L) to IV ferric derisomaltose or usual care [55]. The primary outcome of recurrent HF hospitalizations and CV death showed a non-significant trend favoring IV iron (RR 0.82, 95% CI 0.66–1.02, *p* = 0.070). However, sensitivity analyses censoring COVID-19 pandemic effects suggested benefit (RR 0.76, 95% CI 0.58-1.00), and subgroup analyses indicated greater benefit in patients with TSAT < 15%, highlighting the importance of accurate ID diagnosis. Consistent with this, AFFIRM-AHF randomized 1,132 patients stabilised after an acute HF admission (LVEF < 50%) with iron deficiency to IV ferric carboxymaltose vs. placebo and showed a near-significant reduction in the primary composite of total HF hospitalisations and CV death (RR 0.79, 95% CI 0.62–1.01, *p* = 0.059), driven by fewer total HF hospitalisations (RR 0.74, 95% CI 0.58–0.94, *p* = 0.013), with no difference in CV death (HR 0.96, 95% CI 0.70–1.32, *p* = 0.81) [56]. 

The HEART-FID trial with ferric carboxymaltose enrolled > 3,000 patients but showed minimal overall clinical benefit (unmatched win ratio 1.10, 95% CI 0.99–1.23, *p* = 0.02 for first component [57]. This neutral result may be attributable to specific design features: approximately 60% of HEART-FID participants had a TSAT of > 20% at baseline, raising questions about whether they had “true” ID. Additionally, the trial used strict “withholding” criteria. Investigators applied the baseline definition of iron deficiency (ferritin < 100 µg/L, or 100–300 µg/L with TSAT < 20%) to determine eligibility for all subsequent iron doses, rather than using separate maintenance targets. This resulted in over 80% of patients receiving no therapy between months 6 and 36. This lack of active treatment during follow-up might have prevented the therapy from exerting its effects, contrasting with other trials that used more liberal maintenance cutoffs [58]. 

Recently, the FAIR-HF2 trial evaluated an aggressive FCM dosing strategy in patients with HFrEF [59], and although it yielded a hazard ratio of 0.79 (95% CI 0.63–0.99; *P* = 0.04) for cardiovascular death or first hospitalization, however, the trial missed statistical significance because this p-value did not satisfy the stricter threshold (*P* < 0.0167) required by the Hochberg procedure to adjust for multiple primary endpoints. The FAIR-HFpEF trial, focused on patients with preserved EF, was terminated early due to recruitment issues but demonstrated a significant 49-meter improvement in 6-MWD and fewer serious adverse events [60]. 

## Real-world implementation of guideline-directed medical therapy

Despite strong evidence supporting quadruple therapy in HFrEF, implementation gaps persist [61, 62]. Contemporary real-world data demonstrate that among patients not at target doses, intolerance (not ongoing up-titration) accounts for only a minority of cases: 31.9% for RASi/ARNIs, 30.9% for BBs, and 23.3% for MRAs [63]. The majority (62.2%, 65.0%, and 64.6%, respectively) were classified as “ongoing up-titration,” indicating opportunities for dose optimization.

The TITRATE-HF registry examined 3,367 HFrEF patients with prospective 12-month follow-up, demonstrating that patients reaching ≥ 50% target doses of foundational therapies had significantly greater LVEF improvement (*p* = 0.023 for BBs, *p* = 0.044 for MRAs) and higher rates of achieving ≥ 10% LVEF improvement at 12 months (67.5% for quadruple therapy) [64]. These data reinforce the importance of systematic dose optimization strategies and highlight the need for quality improvement initiatives to close the implementation gap.

## Remote hemodynamic monitoring: pulmonary artery pressure-guided management

The CardioMEMS HF system, measuring pulmonary artery (PA) pressures via implanted sensor, demonstrated benefit in the landmark CHAMPION trial [65]. Since 2021, additional evidence has emerged validating this approach in diverse populations. The MONITOR-HF trial enrolled 348 patients with NYHA class III HF randomized to PA pressure-guided management or standard care [66]. At 12 months, the CardioMEMS group demonstrated significant improvements in quality of life (KCCQ overall summary score difference 8.2 points, *p* < 0.001) and reduced HF hospitalizations (HR 0.63, 95% CI 0.43–0.94). In real-world, non-RCT settings, CardioMEMS has also been shown to lower PAP, improve functional status and quality of life, and reduce HF hospitalizations at 1 year [67]. 

The PROACTIVE-HF trial validated the Cordella sensor, which combines PA pressure monitoring with vital sign tracking [68]. It demonstrated safety and efficacy in guiding decongestion, with low rates of sensor failure and high patient compliance. These trials establish that remote PA pressure monitoring is effective in reducing HF hospitalizations and improving quality of life in appropriately selected patients.

## Transcatheter edge-to-edge repair: from mitral to tricuspid valve

The COAPT trial had established TEER efficacy for secondary mitral regurgitation (MR) in HFrEF [69]. Five-year extended follow-up data demonstrated sustained benefits with TEER plus GDMT vs. GDMT alone [6]. At 5 years, all-cause mortality was 57.3% with TEER vs. 67.2% with GDMT alone (HR 0.72, 95% CI 0.58–0.89), and HF hospitalizations remained significantly reduced (45.2 vs. 67.9% per patient-year, HR 0.53, 95% CI 0.41–0.68). Patients in the TEER group spent an average of 229 additional days alive and out of hospital over 5 years [6]. 

The RESHAPE-HF2 trial provided additional clarity on functional MR in HFrEF [70]. In patients with moderate-to-severe functional MR and symptomatic HF, mitral TEER plus optimal medical therapy significantly reduced the composite rate of CV death or HF hospitalization vs. medical therapy alone (37.0 vs. 58.9 events per 100 patient-years, rate ratio 0.64, 95% CI, 0.48–0.85, *P* = 0.002).

TR is a frequent and debilitating comorbidity in HF, often leading to right ventricular (RV) failure. The TRILUMINATE Pivotal trial evaluated TEER for severe TR using the TriClip system [7]. One-year results showed substantial improvement in quality of life (KCCQ scores increased by 12.3 points more than control), but did not initially show a mortality or hospitalization benefit. However, in a prespecified secondary analysis of the 2-year follow-up, tricuspid TEER was associated with a significant 28% relative risk reduction in HF hospitalizations vs. medical therapy alone [7]. 

## Interatrial shunt devices

The concept of an inter-atrial shunt (IAS) as a self-regulating device to lower left atrial pressure and alleviate HF symptoms and prognosis has been explored in several clinical trials [71–75]. The RELIEVE-HF trial, a sham-controlled RCT, delivered neutral results for the overall population [76]. Critically, a distinct signal of harm with increased mortality and HF events was observed in the HFpEF subgroup, whereas HFrEF patients appeared to benefit. This dichotomy highlights the hemodynamic complexity of HFpEF. This trial likely pauses widespread adoption of interatrial shunts for HFpEF, while any observations in HFrEF should be considered exploratory and require dedicated, adequately powered trials.

## Conclusion

HF management has progressed significantly since the last guidelines, supported by substantial new evidence. The treatment of HFpEF has advanced with the validation of non-steroidal MRAs and incretin-based therapies, providing effective options for this population. In acute heart failure, the standard of care has shifted from symptomatic relief to the rapid, in-hospital optimization of guideline-directed medical therapy. Together with improvements in transcatheter valve interventions and remote monitoring, these developments require an update to clinical practice. Moreover, several clinical trials are currently ongoing, and a selection of these is presented in Table 1. The anticipated 2026 guidelines will likely integrate these advances, emphasizing a personalized approach to ensure the timely implementation of life-saving therapies for all patients.

**Table 1:** Overview of selected ongoing clinical trials for heart failure: summary of key characteristics

Study Name	NCT ID/ Trial Phase	Intervention vs. Control Arms	Population	Primary Endpoint
BalanceD-HF	NCT06307652/ Phase 3	Intervention: Balcinrenone (15 mg or 40 mg) + Dapagliflozin (10 mg) Control: Placebo + Dapagliflozin (10 mg)	HF + CKD (eGFR 20–60) Recent HF event	Time to first occurrence of CV death or HF event (hospitalization or urgent visit).
Prevent-HF	NCT06677060/ Phase 3	Intervention: Baxdrostat + Dapagliflozin Control: Placebo + Dapagliflozin	HF risk factors (T2D, HTN, CVD) + CKD	Time to first occurrence of hospitalization for HF, HF event without hospitalization, or CV death.
REDEFINE-HF	NCT06008197/ Phase 3	Intervention: Finerenone Control: Placebo	HFmrEF/HFpEF post-acute discharge (LVEF ≥ 40%)	Composite of total (first and recurrent) HF events and CV death.
CONFIRMATION-HF	NCT06024746/ Phase 3	Intervention: Finerenone + Empagliflozin (Early Intensive) Control: Usual Care	Hospitalized Acute HF (Any LVEF)	Hierarchical Composite (Win Ratio): All-cause mortality, Total HF events, Time to first HF event, KCCQ-TSS.
FINALITY-HF	NCT06033950/ Phase 3	Intervention: Finerenone Control: Placebo	HFrEF intolerant/ineligible for steroidal MRAs	Time to first occurrence of CV death or HF event.
MARITIME-HF	NCT07037459/ Phase 3	Intervention: Maridebart Cafraglutide (MariTide) Control: Placebo	HFpEF + Obesity	Time to first occurrence of CV death or HF event (hospitalization or urgent visit).
COMET-HF	NCT06736574/ Phase 3	Intervention: Omecamtiv Mecarbil Control: Placebo	Severe HFrEF (LVEF < 30%)	Time to first event of CV death, HF event, LVAD, Heart Transplant, or Stroke.
HERMES	NCT05636176/ Phase 3	Intervention: Ziltivekimab (IL-6 inhibitor) Control: Placebo	HFpEF + Elevated CRP (> 2 mg/L)	Time to first occurrence of CV death, HF hospitalization, or urgent HF visit.
ALT-FLOW II	NCT05686317/ N/A	Intervention: APTURE Interatrial Shunt Control: Sham Procedure	HFpEF / HFmrEF (LVEF > 40%)	Safety: MACCRE at 30 days. Effectiveness: Change in Exercise PCWP at 6 months.
CORCINCH-HF	NCT04331769/ N/A	Intervention: AccuCinch System + GDMT Control: GDMT alone (Open Label)	HFrEF (LVEF 20–40%)	Safety: Freedom from device-related MAEs (6mo). Efficacy: Hierarchical Composite (Win Ratio) of Death, LVAD/Tx, HF Hosp, KCCQ (12mo).
AIM HIGHer	NCT05064709/ N/A	Intervention: CCM (Optimizer Smart Mini) Control: Sham (Device implanted but OFF)	LVEF 40–70% (HFmrEF/HFpEF)	Part 1: Change in 6MWD & KCCQ (6mo). Part 2: Hierarchical composite (Win Ratio) of CV Death, HF Hosp, Urgent visits (18mo) & KCCQ (12mo).
BioVAT-HF	NCT04396899/ Phase 1/2	Intervention: EHM Patches (Stem Cell) Control: None (Single arm)	Terminal HFrEF (LVEF ≤ 35%)	Efficacy: Change in Heart Wall Thickness/Thickening (12mo). Safety: Adverse Events.
REVERSE-HF	NCT05318105/ N/A	Intervention: Aquadex Ultrafiltration Control: IV Loop Diuretics	Diuretic Resistant HF (Fluid Overload)	Win Ratio of CV Mortality, HF events, and Quality of Life within 30 days.

*6MWD, 6-minute walk distance; CCM, cardiac contractility modulation; CKD, chronic kidney disease; CRP, C-reactive protein; CV, cardiovascular; CVD, cardiovascular disease; eGFR, estimated glomerular filtration rate; EHM, engineered heart muscle; GDMT, guideline-directed medical therapy; HF, heart failure; HFmrEF, heart failure with mildly reduced ejection fraction; HFpEF, heart failure with preserved ejection fraction; HFrEF, heart failure with reduced ejection fraction; HTN, hypertension; IL-6, interleukin-6; IV, intravenous; KCCQ, Kansas City Cardiomyopathy Questionnaire; KCCQ-TSS, Kansas City Cardiomyopathy Questionnaire Total Symptom Score; LVAD, left ventricular assist device; LVEF, left ventricular ejection fraction; MACCRE, major adverse cardiac, cerebrovascular, and renal events; MAEs, major adverse events; MRA, mineralocorticoid receptor antagonist; NCT, National Clinical Trial; PCWP, pulmonary capillary wedge pressure; T2D, type 2 diabetes; Tx, transplant.*

## Data Availability

No datasets were generated or analysed during the current study.
